# Supplementary material for: Contrasting effects of information sharing on common-pool resource extraction behavior: Experimental findings
Source: PLoS One. 2020 Oct 7;15(10):e0240212. doi: 10.1371/journal.pone.0240212 (PMC7540899; doi:10.1371/journal.pone.0240212)
Supplement: S1 Appendix — (DOCX) [file pone.0240212.s001.docx]

### S1 Appendix

**Instructions for the experiment (Translated from French)**

The experiment in which you are going to participate is part of a study on decision-making. Please read the instructions carefully. These instructions are meant to help you to understand the experiment. Once all the participants have read the instructions, the experimenter will then reread the instructions aloud. Your gains will depend on your decisions and on the decisions of the other participants. All your answers will be anonymous and will be gathered via a software program. You will indicate your choices on the computer in front of you, and this computer will calculate the gains you have made in the course of the experiment. The amount corresponding to your earnings during the experiment will be paid to you in cash at the end of the experiment. From this moment on, we ask you to refrain from speaking. If you have a question, please raise your hand and an experimenter will help you in private.

Your gain for the experiment will be expressed in experimental currency units (ecus). The conversion rate from ecus to euros is: 1 ecu = 0.02 euro.

**General framework**

At the beginning of the experiment, the central computer will create groups of four members at random. The composition of the groups will remain unchanged for the duration of the experiment. You will not be able to identify the other members of your group and they will not be able to identify you.

20 rounds will be played. At the beginning of each round, the collective account has an endowment of 40 tokens. Every member of the group must decide how many tokens they extract from the account (a number between 0 and 10) to be put into their individual account.

**Functioning of the accounts and earnings**

***Individual account***

Every token you put into your individual account will correspond to 3 ecus for you.

*Example 1*: You extract 4 tokens from the collective account and put them into your individual account. Your gain from your individual accounts corresponds to 12 ecus.

***Collective account***

Each token extracted from the collective account provokes a loss to all members of the group. This loss depends on the total number of tokens, X, extracted by the group from the collective account. The loss suffered by each member is equal to 0.01875 x X2 ecus.

*Example 2*: Your group extracts in total 10 tokens. The loss suffered by each member of the group is 0.01875 x 102 ecus.

**Payoff**

The payoff is equal to 30 ecus plus the sum of the gains from the individual account, minus the loss arising from the collective account.

*Example 3*: You extract 4 tokens from the collective account, and in total your group extracts 10 tokens from this account. Your gain is equal to 30 + 3 x 4 – 0.0875 x 102 = 40.125 ecus, rounded up to 40.13 ecus.

In order to simplify your calculations, you dispose of a table (figure 1) giving you directly your payoff as a function of the number of tokens that you have extracted (columns) and of the total number of tokens extracted by your group (rows). You can find the value of 40.13 ecus of example 3, where your extraction is 4 and the total extraction of the group is 10.


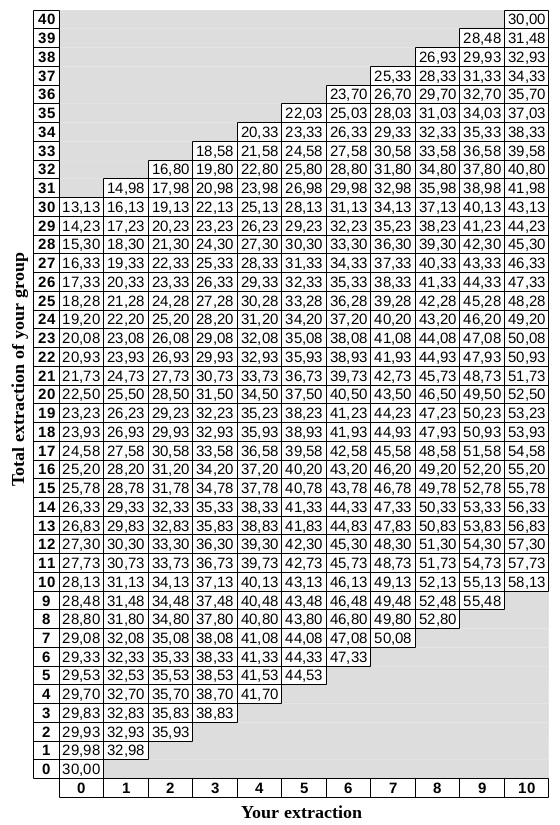


*Figure 1*

***Below we provide the course of one round for each treatment, but a subject participated in only one treatment.***

**Steps of one round (treatment MD)**

Two steps compose one round.

*Step 1: Extraction decision (figure 2)*

In this step, you must decide the number of tokens that you extract from the collective account to put them into your individual account. You can extract as many tokens you want between 0 and 10 tokens. When all participants have taken their decision, the second step is launched.


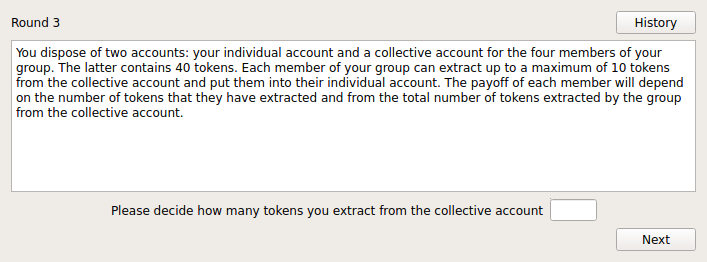

Figure 2

*Step 2: Summary (figure 3)*

This step provides you with information about the number of tokens that you have extracted and your payoff, and informs you about the total number of tokens extracted by your group. You are also informed about the number of tokens extracted by each member of your group. Those numbers are displayed on your screen in a random sequence to ensure anonymity.


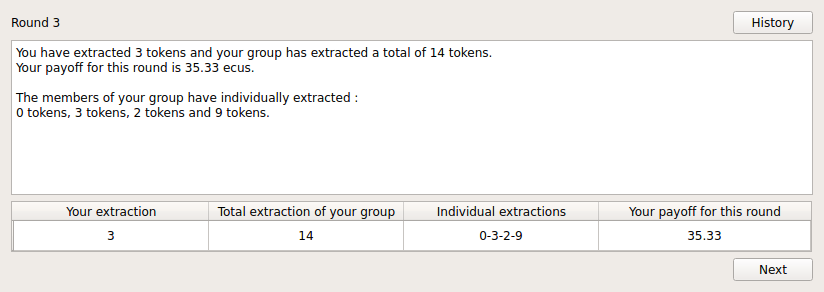
*Figure 3*

**Steps of one round (treatment VD)**

Three steps compose one round.

*Step 1 is the same as in treatment MD.*

*Step 2: Disclosure of information decision (figure 4)*

In this step, you must decide whether or not you want the number of tokens that you have extracted to be shown on the screen of the other members of your group at step 3.

When all participants have taken their decision, the third step is launched.


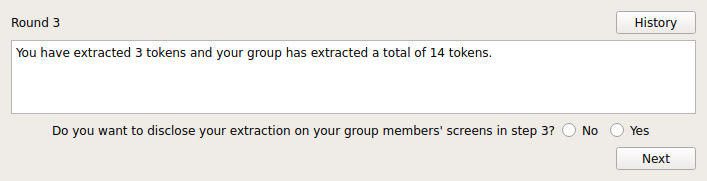
*Figure 4*

*Step 3: Summary (figure 5)*

This step provides you with information about the number of tokens that you have extracted and your gain, and informs you about the total number of tokens extracted by your group. You are also informed about the number of tokens extracted by each member of your group who agreed to disclose this information in step 2. Those numbers are displayed on your screen in a random sequence to ensure anonymity.


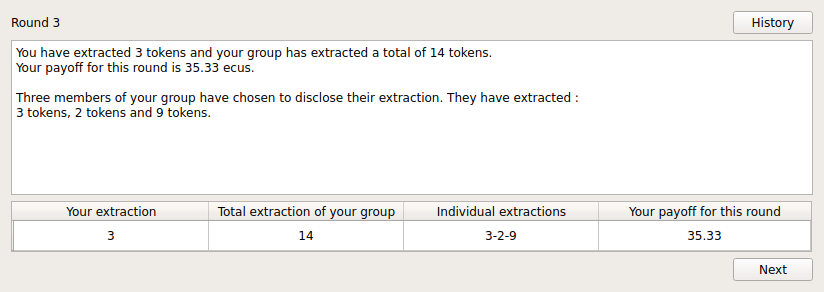
*Figure 5*

**Steps of one round (treatment FD)**

Three steps compose one round.

*Step 1 is the same as in treatments MD and VD.*

*Step 2: Disclosure of information decision (figure 6)*

In this step, you must decide whether or not you want the number of tokens that you have extracted to be shown on the screen of the other members of your group at step 3. If you choose ‘yes’, you must then choose the amount to be shown. By default, this amount corresponds to the number of tokens that you have really chosen to extract, but you can change this number. You are free to show the number you want, as long as it is included between 0 and 10.

When all participants have taken their decision, the third step is launched.


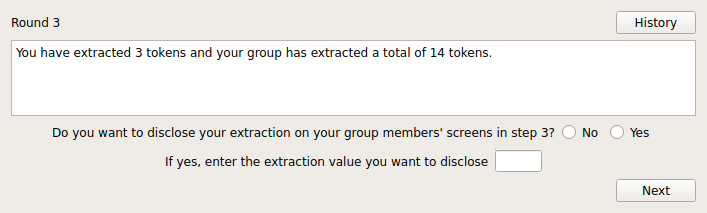
*Figure 6*

*Step 3 is the same as in treatment VD.*

**History**

On each screen you will be able to access the history of past periods by clicking on the “History” button. This history is a table that shows, for each past round, the row of the table displayed on the screen at step 3. An additional column gives you your cumulative payoff from the first round to the current one.

**Final payoff**

Your payoff for this experiment will be equal to your cumulative payoff over the 20 rounds of play. It will be converted into euros and given to you in cash.
