# Supplementary material for: Contrasting effects of information sharing on common-pool resource extraction behavior: Experimental findings
Source: PLoS One. 2020 Oct 7;15(10):e0240212. doi: 10.1371/journal.pone.0240212 (PMC7540899; doi:10.1371/journal.pone.0240212)
Supplement: Attachment — Submitted filename: Report on PONE-D-20-03051.pdf [file pone.0240212.s002.pdf]

Report on

"Contrasting effects of information on CPR extracting behavior: experimental findings"

Submitted for publication in *PLoS ONE*

(Manuscript ID: PONE-D-20-03051)

The paper studies the relative efficiency of three information disclosure mechanisms on common pool resources (CPR) problems: Mandatory disclosure, voluntary disclosure and free disclosure. For this purpose, the authors run an experiment on CPR where groups of 4 individuals decide on the number of tokens (from 1 to 10) they extract, taking into account that their payoffs increase with the tokens they get, but also that there is a negative externality to the group increasing with the total number of tokens extracted in the group. Although the symmetric equilibrium of the resulting game would imply to extract 5 tokens each, the average number of extractions during the 20 rounds is between 7 and 10. These extractions are higher when a mandatory disclosure on the extractions is implemented than when voluntary mechanisms are considered, although the number of extractions increase and converge to the maximum along the experiment in all treatments. However, the results show that there is a significant number of 'cooperative' people that decided to disclose their decisions, which is higher in voluntary than in free disclosing treatment, since the latter tend to exploit the disclose information strategically.

Although the results of the are somehow expected, the experiment is appealing, apparently well-executed and the analysis well-performed, which are the main items for publication in PLoS ONE. However, I also believe that there are several issues that should be considered in the revision. See my main comments below:

Comments

1) There is a paper on CPR published in PLoS ONE (Lacomba et al. 2017) that shows that there is a significant number of cooperators (which decide to follow a sustainable rule of extractions) independently on the disclosure of information about the actions of other players. However, the (mandatory) disclose of information either accelerates the non-cooperative behavior or lead to a more egalitarian share of the resources. These two dynamics are highly dependent on the first choice of the players. This story is also found in the paper under revision, where the initial decisions have a positive

and significant impact in the voluntary contribution treatment. Nevertheless, these ideas are not exploited enough in the paper. I suggest to study all these issues in depth.

2) Linked to the previous comment, I miss a baseline treatment where there is no disclosure of information at all. Would all subjects behave as free-riders or even in this case there would be a significant proportion on cooperators?

3) From the average results in Figure 2 the people that follow the optimal strategy (taken just 5 tokens) cannot be appreciated. It would be interesting an analysis on the percentage of people who follow the 'optimal strategy' in all treatments. I have the impression that subjects are not capable of deducing their optimal strategy from the payoff function in the experiment. Maybe they should have received some examples in the instructions to clarify it. I think that the instructions of the experiment should be appended in the paper to shed light on this issue.

4) The results seem to be highly dependent on the number of people in the groups. For two individuals (as in the paper mentioned in comment #1) free-riders are immediately detected with the disclosure of information. With four subjects, free riders (and information manipulators) can be identified along the experiment and this explains the convergence of voluntary and free disclosure trends. An analysis of the sensibility of results to the number of subjects would be performed or at least mentioned as a possible limitation.

5) It is not clear to me if the students were economically incentivized or not (for instance Conjecture 1 states "Even without a specific monetary incentive"). The experimental economics literature is very critical with non-monetary-incentivized experiments. Therefore, this issue should be also clarified. In general, I miss more details on the experimental procedures, as well as the instructions.

6) The paper run panel data regressions to investigate on the individual decisions on extractions. I would devote a particular Section for the econometric modeling. The models and the variables used should be properly justified, as well as the employed method (a tobit model with correlated random effects). Note that dynamic panel data models, by construction, present endogeneity problems that should be taken into account (maybe through GMM estimation). The group effects have to be treated as well, since every group has different interactions. All the tables can be gathered in a single table so as estimates can be easily compared and statistics on the model performance should be also displayed. As a matter of fact, the interpretation of the results deserves further explanations.

7) It would be also interesting to analyze gender issues in relation to free-riding, voluntary discussing and manipulation on reported information in free disclosure treatment. Are any significant differences between men and women?

8) The paper states that the free disclosure mechanism seems to be less efficient than voluntary disclosure, although less expensive since it does not require an agency for data collection. Nevertheless, it would require an external audit to manage with the strategic manipulation of data. An alternative solution would be the implementation of ‘strategy-proof’ mechanisms that incentivize truth-reporting. I suggest to look for alternative payoff functions where the penalty be less sensitive to the deviations from the optimal strategy (maybe in terms of *N* times de median extraction) or where an extra penalty for randomly detection for liars be introduced.

References

Lacomba, J.A., Lagos, F., Perote, J. (2017). The Lazarillo's game: Sharing resources with asymmetric conditions. *PLOS ONE* 12/7, e0180421.
