# Supplementary material for: Contrasting effects of information sharing on common-pool resource extraction behavior: Experimental findings
Source: PLoS One. 2020 Oct 7;15(10):e0240212. doi: 10.1371/journal.pone.0240212 (PMC7540899; doi:10.1371/journal.pone.0240212)
Supplement: Attachment — Submitted filename: PONE-D-20-03051_review.pdf [file pone.0240212.s003.pdf]

Referee report on manuscript title "Contrasting effects of information sharing on CPR extraction behaviour: experimental findings"

General comment

This paper experimentally addresses the impact of the information-sharing mechanism in a common pool resource (CPR). The authors' primary objective is to find an efficient and low-cost mechanism for the provision of CPR by analyzing the extraction behavior of the subjects through laboratory experiments. The results show that extraction behavior is affected by the mechanism of information sharing about the personal extraction units. Authors have made a comparison between three mechanisms, i.e., (i) a mandatory information disclosure, (ii) a voluntary information disclosure, and (iii) a free information disclosure. Authors claim is that voluntary information sharing mechanism is better than the mandatory information-sharing mechanism; however, giving extra freedom in terms of voluntary information sharing reduces the positive impact of information-sharing mechanism. I have found this research question to be exciting, and it has importance. Information sharing provision in CPR is a very crucial part, and how it affects people's behavior is of great interest to many researchers. However, in general, the current existing manuscripts have several critical shortcomings, which have primary and extra few minor concerns. The authors must address them adequately for publication in any scientific, academic journals. Firstly, the introduction part needs to be adjusted by treating several existing literature to confirm the noble contribution of this paper. The authors have referred literature but treated them in a very superficial manner, and that makes it hard to understand what contribution the paper is trying to make and what theory do authors want to support. Second, the authors failed to clarify how the voluntary information disclosure mechanism can be useful as the accountability for the mechanism is unclear, and unfortunately, authors left even without addressing or discussing them. Third, results are explained in a very superficial manner without any in-depth analysis of the covariates. I could not observe any statistical and economic significance of the regression results, and further, there is no discussion with comparison to the past literature. It is tough to understand the contribution of the current work. Authors can do a much better job in the discussion part by comparing their findings with previous literature to strengthen their findings and provide further insight. The conclusion sections of the paper could have been used to emphasize the value of the exercise that authors have undertaken and the contribution to the literature, however, it is done superficially.

Major comments

1. **Introduction:** The First paragraph of the introduction is not clear. Please see below for specific comments about the first and second paragraphs. In the second paragraph, authors write "The system provides the company with more precise and timely information on the water consumption of irrigators with a double objective of more efficient water allocation and making sure that the quotas allocated to users are respected. In general, even when well established like the water and electricity utilities described here, most regulatory agencies lack information on user consumption and users have little information on the stock of resources and its consumption by others." The system ? what kind of system or which system authors want to address here and there is no citation of the source? These two sentences are not clear. In the introduction, the first paragraph can give general background and the second paragraph can be more specific with more details so that it gives readers an idea about the

issue that authors are going to deal with in their manuscript. I also feel the second paragraph is too long and some redundancy that needs to be reduced and improved.

2. Page 3, third paragraph, authors write "In some countries, like the United States, India or the Maghreb among others, groundwater irrigation has developed rapidly, causing problems with the depletion of aquifers and the sustainability of these systems." "These system" is not apparent here in this sentence, what system authors want to address they need to be specific, groundwater irrigation system? The objective of the third paragraph is not clear, and it is better to summarize with one sentence by the end of the paragraph and to be followed by the open question; therefore, readers can understand.

3. Page 3, fourth paragraph, a sentence such as "Several recent articles focused on information, and this is also the case in our article." Please explain in more detail what previous literature has already worked on by focusing on information provision and what is new in this research. Otherwise, give details on what others have done and how it is different from your experiments. Last sentence is not clear please elaborate "This result is also observed when the subjects are placed in a long-term context (Huck et al. 2017)."

4. Page 4 and 5, in fifth and sixth paragraph authors, have mentioned about static common-pool resources then it might also make sense to discuss dynamic common pool resources and further explain and justify why the author decided to chose static nature of CPR as many CPR are dynamic?, for example, see Timilsina et al. (2017). (Saijo et al., 2017; Timilsina et al., 2017; Hauser et al., 2014) are some other articles not appropriately treated if authors are discussing the resource utilization and its correlation with prosociality and stability of the mechanism. Framing effect (negative framing) in CPR lab experiment is rare, but Timilsina et al. (2017) is one of the few works that is published in "Plos One" and have used such negative frame, but the authors have missed this literature to discuss, please addressed these issues while conducting the literature review.

Experimental design and Conjecture:

5. Please, explain the experimental procedure in more detail so that readers can fully understand and visualize the experiments. If possible, a flow-chart could be helpful for an explanation.

6. Authors can explain the treatments and practical procedure more clearly, such that what happens in the first stage and second stage and so on. It is hard for readers to understand. In MD treatment, authors write, "This was our baseline treatment. In this treatment, players were informed of each extraction decision made by the other members of their group. Individuals' extractions were anonymous, meaning that values were displayed without any ID attached. This information was provided in summary so that there were only two steps in this treatment (extraction decision and summary)." Please explain what subjects do in this treatment without making a logical jump, so that readers can understand. It is confusing in a sense that what subjects do, can they see other's extraction information before they make their own decision, or after they make their decision first, it is not clear.

7. Authors explain VD as "In this treatment; each player had to decide, in the second stage of the period, whether they wished their extraction decision to be made public in their group.

If so, the extraction level of the player was made public in the group. Specifically, it was anonymously displayed in the summary screen for the period of each member of their group. If the player refused, the information was not displayed." Please, explain what happens in the first stage of the period. The second sentence uses, "If so" this is an informal expression in academic writing, it is better to reduce such expression if possible.

8. Author explains FD "However, in the case of disclosure, the player chose the amount that was made public. In other words, compared to VD, the player was free to choose the extraction level that was displayed on the screen of the others. The player might, therefore, decide to disclose an amount different from the actual extraction level." Please, explain this procedure in more detail. In my understanding of FD treatment, subjects have the opportunity to lie about their extraction decision. If that is true, then please indicate such information. Also, decision-making steps need to be precisely given or explained.

9. Instructions are not included in the appendix, and it might help to understand experiments in more detail. Please include the experiment instruction in the appendix. Economic experiments need external validity and replication; sometimes, for that purpose, it is essential to include experimental instruction.

Conjectures:

10. "Conjecture 1: Even without a specific monetary incentive to do so, many players voluntarily choose to disclose their extraction decision to their group members. This is a necessary condition for the mechanism's efficiency to be tested." It is unclear here what kind of condition that *"This is a necessary condition"* refers. Consider not to use such unclear antecedents. Authors could also provide further details on the background why they made such conjecture, and also provide hints to the readers what kind of prediction they have beforehand.

11. "Conjecture 3.1, 3.2" I could not find proper support for these two conjectures. I am still wondering what the underlying basis for the free disclosure treatments, where people are free-riders, how their behavior can be corrected utilizing such treatment. The authors need to provide a full description of the justification of why they have used this treatment. Uncontrolled freedom is chaos and not efficient, and it is self-evident. If the authors have chosen this treatment, then there should be a reliable, valid reason for that. However, in the current manuscript, it is entirely missing. Furthermore, the authors need to pay extra attention to clarify their objectives, are they trying to see if allowed to lie how it affects people behaviors to manage CPR or social learning effect of a lie? Please explain.

Results:

12. First paragraph of the Results section, in the results section, authors begin to discuss the treatment effect that is too sudden. It might be better to start by explaining summary stats of the pre and post-treatment covariates, and also, it is better to show how the pre-treatment covariates do not differ across treatment; therefore, the authors can claim that result that mainly derived is only from the treatment effects.

13. In page 10, results from Figure 1, is not very informative. It is better to show the frequency of disclose per rounds.

14. Table 2 should come before figure 1 while discussing summary statistics at the beginning of the results section and also please include sample size number in table 2.

15. results section the third paragraph is very long, and it should be broken down into two parts, the first paragraph for describing the technical aspect of the model next for explaining the economic and statistical significance. In table three, it is more informative if authors could report marginal effects.

16. Tables 4, 5, and 6, please report marginal effect, and discussion is necessary to compare with other past literature how the results are similar and are different from the past work. Please add the discussion section. For example, information sharing and the non-lying previous period dummy is significant what does that mean please provide further explanation.

17. One more thing is that authors have discussed the prosociality, and conditional cooperators in conjecture but have failed to discuss in the results sections. Why authors did not control for the prosociality of the people, to characterize what kind of people follow VD mechanism is essential to understand. If these issues are out of scope of the current experiments, then please explain and mention it in the limitations of the current work.

    Conclusion:

18. Authors write "Allowing agents to self-report is a mechanism that would likely benefit from the addition of an external audit system. Such an audit system would reduce the observed strategic behaviour and thus make the FD mechanism both more efficient and less expensive than other types of procedures." This is more-like a limitations of this paper. There is no audit system that this experiments have checked. Therefore, please write what are the pure limitation of the current work.

19. Policy implication can be provided in conclusive way but the current manuscript is lacking that. Please, clearly mention what kind of policy implication can be drawn for this research.

Specific comments

1. In the abstract authors write, "Our main result was that the mechanism of voluntary information sharing could help in reaching a lower average extraction level than that observed with the mandatory mechanism. However, giving actors more freedom in terms of voluntary information sharing introduced strategic considerations that could lessen this positive impact." Because it is academic paper and results, and it is highly recommended to be expressed in the present perfect tense rather than the past tense. Also, results can be more expressed in scientific terms with statistical reference along with the magnitude of the effects if possible rather than making it general.

2. Page 2, 1st paragraph seems to be very short, and what authors want to express is not clear. It seems the authors are trying to set-up a background for their research question, but it does not fulfill the author's objective. It needs to be re-written. If authors want to set-up a global picture as a background, then it needs to supported by past literature. It is challenging for readers to understand what problems are authors going to address in a current state?

3. Page 2, 2nd paragraph, “In the electricity sector, for example, many countries have deployed smart grids to place data flow and information management at the heart of improving supply efficiency.” is this general statement or finding of some past research? where is the source?

4. Similarly, “In the case of domestic water use, particularly in Western countries, remote metering enables water services and providers to improve the efficiency of water supplies by reducing costs and leaks.” What does it mean by “ reducing costs and leaks” is it empirical work? If it is, then please cite the source. The same goes for the sentence “More real-time information on consumption is also provided to water users, who can then adjust their habits and detect possible leaks or abuse, thereby reducing their water bill.”Please cite source.

5. Abbreviation has been used without defining it, as authors write “In southwestern France, for example, the local water management company (CACG) operates a system for remote reading of irrigation water consumption.” CACG?

Minor comments

- The manuscript contains several typos and odd phrasing throughout that need to be addressed

Overall evaluation

The main question in this research is compelling. However, there are several issues and biases that authors need to handle to overcome the shortcomings of this paper. It appears that authors have taken several things in a very light manner without paying enough attention. The current experiments could not fully convince the readers how the mechanism of voluntary information disclosure can be implemented in real life. However, it is a new issue that authors are trying to handle, but it is crucial to be clear what the authors can claim and cannot form the current experiments. It is advisable to be clear on the part “scope of experiments” and to state the limitations and future work if something is out of scope. Therefore, to publish this paper, the authors need to handle the significant issues raised seriously. Moreover, authors should also keep in mind that this work is going to be published in the multidisciplinary journal. Therefore, policy implication needs to clear cut for the general readers.

References

Hauser, O. P., Rand, D. G., Peysakhovich, A., and Nowak, M. A. (2014). Cooperating with the future. 511:220–223.

Saijo, T., Feng, J., and Kobayashi, Y. (2017). Common-pool resources are intrinsically unstable. *International journal of the commons*, 11:597–620.

Timilsina, R., Kotani, K., and Kamijo, Y. (2017). Sustainability of common pool resources. 12:e0170981.
