# Supplementary material for: Contrasting effects of information sharing on common-pool resource extraction behavior: Experimental findings
Source: PLoS One. 2020 Oct 7;15(10):e0240212. doi: 10.1371/journal.pone.0240212 (PMC7540899; doi:10.1371/journal.pone.0240212)
Supplement: Attachment — Submitted filename: revision_reponse_reviewer_4_1.docx [file pone.0240212.s004.docx]

**Reviewer 4**

***Introduction: The First paragraph of the introduction is not clear. Please see below for specific comments about the first and second paragraphs. In the second paragraph, authors write “The system provides the company with more precise and timely information on the water consumption of irrigators with a double objective of more efficient water allocation and making sure that the quotas allocated to users are respected. In general, even when well established like the water and electricity utilities described here, most regulatory agencies lack information on user consumption and users have little information on the stock of resources and its consumption by others.” The system ? what kind of system or which system authors want to address here and there is no citation of the source? These two sentences are not clear. In the introduction, the first paragraph can give general background and the second paragraph can be more specific with more details so that it gives readers an idea about the issue that authors are going to deal with in their manuscript. I also feel the second paragraph is too long and some redundancy that needs to be reduced and improved.***

***Page 3, third paragraph, authors write “In some countries, like the United States, India or the Maghreb among others, groundwater irrigation has developed rapidly, causing problems with the depletion of aquifers and the sustainability of these systems.” “These system” is not apparent here in this sentence, what system authors want to address they need to be specific, groundwater irrigation system? The objective of the third paragraph is not clear, and it is better to summarize with one sentence by the end of the paragraph and to be followed by the open question; therefore, readers can understand.***

***Page 3, fourth paragraph, a sentence such as “Several recent articles focused on information, and this is also the case in our article.” Please explain in more detail what previous literature has already worked on by focusing on information provision and what is new in this research. Otherwise, give details on what others have done and how it is different from your experiments. Last sentence is not clear please elaborate “This result is also observed when the subjects are placed in a long-term context (Huck et al. 2017).”***

The introduction has been largely rewritten, taking care to quickly present the context, the research question and the relevant literature. The paragraph containing the examples has been shortened.

***Page 4 and 5, in fifth and sixth paragraph authors, have mentioned about static common pool resources then it might also make sense to discuss dynamic common pool resources and further explain and justify why the author decided to chose static nature of CPR as many CPR are dynamic?, for example, see Timilsina et al. (2017). (Saijo et al., 2017; Timilsina et al., 2017; Hauser et al., 2014) are some other articles not appropriately treated if authors are discussing the resource utilization and its correlation with prosociality and stability of the mechanism. Framing effect (negative framing) in CPR lab experiment is rare, but Timilsina et al. (2017) is one of the few works that is published in “Plos One” and have used such negative frame, but the authors have missed this literature to discuss, please addressed these issues while conducting the literature review.***

You are right, as Timilsina et al (2017) point out, much of the literature on common-pool resources is based on static repeated games. We are ourselves in this literature, and more specifically in the branch that is concerned with the provision of social information. The implementation of the mechanisms we propose in a dynamic environment is a very good idea for extension. However, for a first case study we believe that the static framework is easier and allows a better understanding of the behavioral dimension of the effects induced by these voluntary sharing mechanisms.

***Please, explain the experimental procedure in more detail so that readers can fully understand and visualize the experiments. If possible, a flow-chart could be helpful for an explanation.***

***Authors can explain the treatments and practical procedure more clearly, such that what happens in the first stage and second stage and so on. It is hard for readers to understand. In MD treatment, authors write, “This was our baseline treatment. In this treatment, players were informed of each extraction decision made by the other members of their group. Individuals’ extractions were anonymous, meaning that values were displayed without any ID attached. This information was provided in summary so that there were only two steps in this treatment (extraction decision and summary).” Please explain what subjects do in this treatment without making a logical jump, so that readers can understand. It is confusing in a sense that what subjects do, can they see other’s extraction information before they make their own decision, or after they make their decision first, it is not clear.***

***Authors explain VD as “In this treatment; each player had to decide, in the second stage of the period, whether they wished their extraction decision to be made public in their group. If so, the extraction level of the player was made public in the group. Specifically, it was anonymously displayed in the summary screen for the period of each member of their group. If the player refused, the information was not displayed.” Please, explain what happens in the first stage of the period. The second sentence uses, “If so” this is an informal expression in academic writing, it is better to reduce such expression if possible.***

***Author explains FD “However, in the case of disclosure, the player chose the amount that was made public. In other words, compared to VD, the player was free to choose the extraction level that was displayed on the screen of the others. The player might, therefore, decide to disclose an amount different from the actual extraction level.” Please, explain this procedure in more detail. In my understanding of FD treatment, subjects have the opportunity to lie about their extraction decision. If that is true, then please indicate such information. Also, decision-making steps need to be precisely given or explained.***

***Instructions are not included in the appendix, and it might help to understand experiments in more detail. Please include the experiment instruction in the appendix. Economic experiments need external validity and replication; sometimes, for that purpose, it is essential to include experimental instruction.***

We agree that the section dedicated to the explanation of the experience was not precise enough, and that instructions were missing. We have translated the instructions, and they are now attached to the article. In addition, we have largely rewritten the corresponding section. The description of the different treatments is clearer, and a table has been added to describe the different steps within a round, for each treatment.

***”Conjecture 1: Even without a specific monetary incentive to do so, many players voluntarily choose to disclose their extraction decision to their group members. This is a necessary condition for the mechanism’s efficiency to be tested.” It is unclear here what kind of condition that “This is a necessary condition” refers. Consider not to use such unclear antecedents. Authors could also provide further details on the background why they made such conjecture, and also provide hints to the readers what kind of prediction they have beforehand.***

You're right. This conjecture is based on Kreitmair's paper (2015). What we meant was that if players do not disclose their extractions then the following conjectures about the expected effects cannot be tested. We have changed the presentation of this conjecture and have provided a better motivation.

***“Conjecture 3.1, 3.2” I could not find proper support for these two conjectures. I am still wondering what the underlying basis for the free disclosure treatments, where people are free-riders, how their behavior can be corrected utilizing such treatment. The authors need to provide a full description of the justification of why they have used this treatment. Uncontrolled freedom is chaos and not efficient, and it is self-evident. If the authors have chosen this treatment, then there should be a reliable, valid reason for that. However, in the current manuscript, it is entirely missing. Furthermore, the authors need to pay extra attention to clarify their objectives, are they trying to see if allowed to lie how it affects people behaviors to manage CPR or social learning effect of a lie? Please explain.***

We agree that from a theoretical point of view, a selfish individual seeking to maximize his gains in FD treatment will voluntarily disclose a lower value than his actual extraction, hoping to take advantage of this false information to increase his personal gains. What we do not know, however, is the impact this may have on group-wide extraction and its evolution over time. As discussed in the conclusion, this mechanism of free voluntary disclosure is more likely to be socially acceptable, both for the collection and dissemination of private information. However, it is still necessary to consider the behavioral effects induced by this mechanism, and to ensure that it does not lead to a quick over-exploitation of the resource. This is our objective in this paper. The next step is to propose mechanisms to improve its effectiveness, such as auditing.

***First paragraph of the Results section, in the results section, authors begin to discuss the treatment effect that is too sudden. It might be better to start by explaining summary stats of the pre and post-treatment covariates, and also, it is better to show how the pre-treatment covariates do not differ across treatment; therefore, the authors can claim that result that mainly derived is only from the treatment effects.***

Sorry, but we are not sure we understand this point about pre and post treatment covariates. Our experimental protocol was of the between-subject type, so each subject participated in only one treatment. Experimental conditions were identical in all sessions (group size, gender, average age, etc.). The only difference was the treatment. Also, all variables used for the analysis were collected during the experiment. We did not delete any data and did not add any new variables for the analysis.

***In page 10, results from Figure 1, is not very informative. It is better to show the frequency of disclose per rounds.***

We have added in the same figure the graph with the evolution of the frequency of voluntary disclosure in the two treatments.

***Table 2 should come before figure 1 while discussing summary statistics at the beginning of the results section and also please include sample size number in table 2.***

Done. We have also added additional information in this table (standard deviation).

***Results section the third paragraph is very long, and it should be broken down into two parts, the first paragraph for describing the technical aspect of the model next for explaining the economic and statistical significance. In table three, it is more informative if authors could report marginal effects.***

***Tables 4, 5, and 6, please report marginal effect, and discussion is necessary to compare with other past literature how the results are similar and are different from the past work. Please add the discussion section. For example, information sharing and the non-lying previous period dummy is significant what does that mean please provide further explanation.***

Following your comment, we split the section into two parts. We also gather estimation results by treatment in a single table (Table 5).

Besides the estimated coefficients, we report in Table 4 (for the whole sample) and Table 5 (results by treatment) the marginal effects (or partial effects) of explanatory variables on the expected value of individual extraction decision when it is censored at 0 and 10.

Based another reviewer’s comment, we also compare our CRE dynamic Tobit model to the standard random effects Tobit model by using a likelihood ratio Chi2 test. The results show unambiguously that the static model is dominated by our model for the whole sample where all the treatments are gathered as well as for each of the treatments (see Tables 4 & 5).

Finally, we run the CRE dynamic Tobit model without control variables (“Decision-making time” and “Time trend”). The signs of the coefficients of our main variables remain unchanged. Moreover, the likelihood-ratio test (which is a Chi(2) distributed under the null hypothesis of the model without control variable) shows that our model is strongly preferred, either for the whole sample or for every treatment (see Tables 4 & 5). Test results are reported in Footnotes 10 and 12 of the revised manuscript.

Besides the estimated coefficients, we report in Table 4 (for the whole sample) and Table 5 (results by treatment) the marginal effects (or partial effects) as described above. These partial effects globally confirm our interpretation regarding coefficients.

We have also added a discussion paragraph on the results of the estimates.

***One more thing is that authors have discussed the prosociality, and conditional cooperators in conjecture but have failed to discuss in the results sections. Why authors did not control for the prosociality of the people, to characterize what kind of people follow VD mechanism is essential to understand. If these issues are out of scope of the current experiments, then please explain and mention it in the limitations of the current work.***

You're right. If we were to repeat this experience, we would test the behavior of players beforehand to find out their type, probably with some of the procedures used by Burlanda & Guala (2005). The Social Value Orientation (SVO) test could also have been useful (Murphy et al. 2011).

The experimental protocol allows us to observe, in the game, which types of players make their decisions public through the amount they extract from the resource. However, it is true that it is difficult to distinguish conditional cooperators from unconditional cooperators. We have added this idea in the possible extensions of this study.

***Authors write “Allowing agents to self-report is a mechanism that would likely benefit from the addition of an external audit system. Such an audit system would reduce the observed strategic behaviour and thus make the FD mechanism both more efficient and less expensive than other types of procedures.” This is more-like a limitations of this paper. There is no audit system that this experiments have checked. Therefore, please write what are the pure limitation of the current work.***

The establishment of an audit system is, in our opinion, a further step. It was important to have an idea beforehand of the effects of the voluntary free disclosure mechanism. Auditing is an accompanying measure to the mechanism, probably allowing it (to be tested) to be more effective by limiting the disclosure of false information for purely strategic purposes.

***Policy implication can be provided in conclusive way but the current manuscript is lacking that. Please, clearly mention what kind of policy implication can be drawn for this research.***

A discussion about the policy implications that can be drawn from this research was added. Particularly, we introduced thoughts about the mechanisms of self-reporting, frequently used in environmental management, and their possible combination with auditing systems that would improve their effectiveness due to non-compliance.

***In the abstract authors write, “Our main result was that the mechanism of voluntary information sharing could help in reaching a lower average extraction level than that observed with the mandatory mechanism. However, giving actors more freedom in terms of voluntary information sharing introduced strategic considerations that could lessen this positive impact.” Because it is academic paper and results, and it is highly recommended to be expressed in the present perfect tense rather than the past tense. Also, results can be more expressed in scientific terms with statistical reference along with the magnitude of the effects if possible rather than making it general.***

The article has been fully proofread and edited by a native English-speaking proof editor. We informed him about your comments, and in the new version we use the present perfect.

***Page 2, 1st paragraph seems to be very short, and what authors want to express is not clear. It seems the authors are trying to set-up a background for their research question, but it does not fulfill the author’s objective. It needs to be re-written. If authors want to set-up a global picture as a background, then it needs to supported by past literature. It is challenging for readers to understand what problems are authors going to address in a current state?***

The introduction has been largely rewritten. We hope that the new version is more explicit on the problematic of the article and its positioning within the literature.

***Page 2, 2nd paragraph, “In the electricity sector, for example, many countries have deployed smart grids to place data flow and information management at the heart of improving supply efficiency.” is this general statement or finding of some past research? where is the source?***

Among other reports, a study by the National Energy Technology Laboratory for the U.S. Department of Energy reported that the Italian ENEL ‘Telegestore’ smart grid project started in 2005 for a 2.1 billion € total investment. The project was producing 500 million € savings per year and delivering better service at lower cost.

The report produced also an economic analysis of the efficiency improvement of a smart grid system for the electricity supply in the US. This report was followed by the U.S. Dept. of Energy's Smart Grid Program funded by the American Recovery and Reinvestment Act of 2009. This is one of the largest smart grid deployment programs in the world to-date.

Ref: National Energy Technology Laboratory (2007). ‘Modern Grid Benefits’, Report for the U.S. Department of Energy, Office of Electricity Delivery and Energy Reliability, August 2007, 19p.

***Similarly, “In the case of domestic water use, particularly in Western countries, remote metering enables water services and providers to improve the efficiency of water supplies by reducing costs and leaks.” What does it mean by “ reducing costs and leaks” is it empirical work? If it is, then please cite the source. The same goes for the sentence “More real-time information on consumption is also provided to water users, who can then adjust their habits and detect possible leaks or abuse, thereby reducing their water bill.” Please cite source.***

Smart grids and smart meters allow collecting and sharing information about water use of all connected users, but also identifying leaks and anomalies on the network. This information makes it possible to optimize the resource management, fix problems quickly, and therefore reduce the cost of water management. Most of these ideas were discussed in the web conference cited in our article:

Suez Eau France (2019), “Le compteur d’eau connecté, un composant de la ville intelligente”, Web Conference organized by Ideal CO and Suez, 17 January 2019.

An interesting synthesis about smart-grid adoption in the French water sector is available at the web page (in French) : <http://www.smartgrids-cre.fr/index.php?p=reseaux-eau-comptage-intelligent>

***Abbreviation has been used without defining it, as authors write “In southwestern France, for example, the local water management company (CACG) operates a system for remote reading of irrigation water consumption.” CACG?***

This has been fixed.
