# Supplementary material for: Contrasting effects of information sharing on common-pool resource extraction behavior: Experimental findings
Source: PLoS One. 2020 Oct 7;15(10):e0240212. doi: 10.1371/journal.pone.0240212 (PMC7540899; doi:10.1371/journal.pone.0240212)
Supplement: Attachment — Submitted filename: revision_2_answers_001.docx [file pone.0240212.s005.docx]

**Reviewer #2**

*The experiment is well-executed, the analysis well-performed and the results represent interesting contributions to behavioral and public economics, which are the main items for publication in PLoS ONE. The paper has significantly improved in the revision as a consequence of the comments of all the reviewers’ comments. Personally, I am fully satisfied with the answers of the authors.*

*Maybe I would also state a minor comment before publication. Please, revise that there is a one-to-one correspondence between cites and references (I have detected that some cited papers are not included in the references, e.g. Lacomba et al., 2017).*

Thank you for your careful reading. We have added the missing references.

**Reviewer #3**

*The paper has much improved but it still requires much work. The authors did not sufficiently address all my points in their revision.*

Thank you for your careful reading. We are grateful for your remarks and suggestions, it has helped to dramatically improve the article.

**General comments**

*1. Information sharing as pro-social act: I am still not convinced that in the context of this experiment, sharing extraction information can be called pro-social in general. On page 4 the authors write that sharing one's decision can also be seen as a pro-social act that enhances one's social image and self image. Note, however, that the motive of enhancing one’s social image and/or self-image does not imply other-regarding preferences.*

You are right, sharing its decision with others is not necessarily per se a prosocial behaviour. But, as we note in the article, cooperative people are more inclined to disclose their extractions, so there is an indirect link between prosocial behavior and the willingness to share its decision with others. To avoid the confusion, we removed the sentence «Our main assumption was that the voluntary sharing of one’s extraction was a prosocial act to signal one’s cooperative intensions to the group ....»

We also removed the sentence you refer to (Sharing one’s decision ...), and added the two following ones: They may also feel satisfaction in showing their cooperation in the social dilemma, the warm-glow effect of Andreoni (1990), and acting in a prosocial manner, that benefits other people (Brief & Motowidio 1986). If the most cooperative individuals voluntarily share their extraction decision with the other members of their group, the social information provided to the group is likely to favor the emergence of a cooperative descriptive norm (Cialdini 1990), as observed by Croson & Shang (2008), and to trigger conditional cooperative behavior (Fischbacher et al. 2001)..

2. *Lab experiments with economic games are not a standard approach in the social sciences and therefore the readership of PLOS ONE may not be entirely familiar with the terminology the authors use. Of course it is not necessary to explain everything from the start. I would only like to encourage the authors to read their paper through the lens of someone who is not an experimental economist and see whether everything is so self-explanatory (it is not).*

We have tried to be more explicit in the explanation of the experimental design and to remove specific terminology where it was not essential. However, you may have specific words or phrases in mind. If so, please let us know what they are and we will make the necessary changes.

*3. The paper would benefit from having it edited by a professional English editing service.*

This was not possible during this summer vacation time, but we engage, if the article is accepted, to send it to a professional english editing service as soon as possible.

*4. If I remember correctly, PLOS ONE does not allow footnotes. The authors should check this and integrate the text they currently have in the footnotes in the main text.*

You are right, thank you. We have integrated the footnotes in the main text.

*5. Throughout the paper, subscript notation does not seem to be working out very well. The authors should take greater care when editing in-text formulas, equations, variables, etc.*

Yes. It is because we work with OpenOffice (*.odt) but the PLosOne submission platform only accepts *.docx format. We saved our document in the required format, but since the original file was an odt, when the platform compiled the pdf the subscripts and equations didn’t render well. For this revision round, we used the wps application, hopping that the compilation will be fine and the problems fixed.

*6. There are a few points regarding the econometric model that are difficult to understand (see my particular comments on that).*

We answer in the particular comments section below.

**Particular comments**

*1. Table 2 should mention in MD at Step 2 that players had no choice. Otherwise one might wonder what happens in this condition. Also in the text the authors should say that nothing happens in Step 2 of MD, and in Step 3 subjects get the round summary. It will be clearer and more consistent in this way.*

We’ve changed the corresponding text. It seems to us that it is clearer now.

*2. It is still unclear how subjects were randomly assigned to experimental conditions (randomization, not random selection, is the quintessential precondition for causal inference in experiments). If randomization was not possible, the authors should mention it explicitly.*

We used the standard procedure in experimental economics. We invited a random subgroup of the pool of volunteers (nearly 3000, managed by the ORSEE platform) to register for one of the planned sessions. A maximum of 20 subjects could participate in a session, the experimental laboratory being composed of 20 boxes. We applied a between-subject design, which means that all participants in a session played the same single treatment (either MD, VD or FD).

*3. On page 12, the sentence Experimental economics is very useful in this case too, does not seem to be very useful.*

We removed it.

*4. Despite of what the authors write in their reply, the use of terminology is still not consistent. For example, in Figure 1, y-axes are labeled with disclosure and sharing although, in all likelihood, these terms are meant to say the same. Speaking of Figure 1 (and the other figures for that matter), the labels are very small, which makes it difficult to read the figures. The authors should try to improve this.*

You're right. As you've noticed, we use both terms, "voluntary disclosure" and "voluntary sharing", to refer to the same idea. This is to avoid too many repetitions in the text. In figure 1, you're right, it wasn't consistent. We have corrected it by putting the same label on the y-axis of the two graphs. We have also increased the font size of the ticks and labels.

*5. Bottom of page 13 and top of page 14, what does statistic = stand for? What kind of statistic do the authors report here (Chi2? F? t?). Were these tests comparing average extractions for all 20 rounds? And, please, do not use one-sided p-values. Use two-sided with alpha = 5% throughout the paper. This is standard (probably also in PLOS ONE).*

The statistic corresponds to the non-parametric Wilcoxon rank-sum test. Following your remark, we now indicate rank-sum test statistic where applicable. These tests compare the data reported in Figure 2 (i.e. averages extractions calculated for each of the 20 rounds considered, thus each series has 20 observations).

We employed one-sided or two-sided p-values depending on the hypothesis we made. More precisely, the comparison between MD and VD treatments (MD > VD) and that between MD and FD treatments (MD > FD) correspond to a one-sided test because our hypothesis is that the average in this treatment is higher than in both treatments for the voluntary sharing procedure. However, the comparison between VD and FD treatments (VD is different from FD) should correspond to a two-sided test.

However as you suggested we now reported two-sided tests, it doesn’t change the statistical significativity of the differences between the treatments.

*6. Concerning the econometric model described on page 14, I was wondering why the authors have a lagged dependent as well as the total group extraction level from the previous round. At least the second variables should be total group extraction minus player i’s extraction. This will reduce collinearity and better allow to identify conditional cooperation.*

Thank you for your suggestion about using the variable total group extraction minus player i’s extraction. You are right by stressing that your variable reduces correlation between variables. Indeed, the correlation between your suggested variable and individual past decision is about 0.37 whereas the correlation between our variable group past decision and individual past decision is 0.67. Fortunately, the latter is much lower than 1, thus the risk of multi-collinearity (where correlation is close to 1) is ruled out.

There is another reason supporting the use of our model. The latter is equivalent to the model using your suggested variable Total group extraction minus player i’s extraction (or in other words the two models are the same). The argument is as follows. First, consider our regression model with includes the following terms: where is the extraction of player i in the previous round and is total group extraction in the previous round (this variable is included in the set of explanatory variables). Our model allows to estimate coefficients and. Now, consider the model with the variable as you suggested, the regression should include the following terms:. Both models are equivalent because we have (hence,) and. Therefore, we can indifferently estimate either one or the other model. More importantly, the coefficient associated to variable Group past decision (with or without player i’s extraction) remains the same (), leading to the same interpretation about conditional cooperation.

*7. What is the theoretical rationale for including decision time in the model. The authors need to justify that (I did not see they do that in the earlier parts of the paper).*

We are used to measuring decision time in our experiments, since several studies have shown that there is a correlation between decision time and the decision itself (Rubinstein 2007). We had not a particular hypothesis about decision time in our experiment. However estimates show that players who make their decision quickly, on average, extract more, i.e. they are less cooperative. This is consistent with the study of Piovesan & Wengstrom (2009) which found that faster subjects more often choose the option with the highest payoff for themselves. Extracting less from the common resource seems to be based on a more cognitive decision-making process. We have noted this in the current revision.

*8. On page 15/16, the authors write: Finally, the estimation showed that, regardless of the treatment, the first decision in the game mattered. However, as far as I can see, this is not something they can conclude based on the estimation of the model shown in Table 4. To be able to say that there is a positive effect of the initial decision irrespective of treatment, there must be no significant interaction effects. But the authors do not estimate interactions between treatment variables and initial decisions. Moreover, they admit that these effects differ across treatment on page 19 (contradicting their initial statement).*

We are sorry for that confusing sentence. Our idea is that the first decision in the game matters, ceteris paribus, i.e. without any condition on the type of treatment. This sentence is now corrected as follows:

Finally, the estimation showed that, other things being equal, the first decision in the game mattered.

*9. Related to that, I was wondering how the authors, in the statistical model, distinguish between the individuals’ past decision in round 2 and these individuals’ initial decision. Maybe I missed in the text the authors’ explanations.*

As our model is dynamic, if the analysis used only the first two rounds (t=1 and 2), there would be a problem of identification. Hopefully, we use the whole set of values corresponding to t=1,2,,20. This problem no longer exists.

*10. In figure 4, I find it interesting that the subjects that lie about their extractions lie increasingly more with the average increase of extraction levels. They seem to believe that once things go to hell, lying more about the true state of nature could maintain things in their favor. I am juts wondering whether the authors would like to say a bit more about this finding in the paper.*

We also found this was an interesting point. However it is no a significant increase of lies and we are not able to draw a conclusion about what happened in their mind.

We have added the following sentences about it : After the tenth round, when the average extraction starts to increase, one can observe a disclosed extraction, by lying players, that decreases. A simple linear regression gives a coefficient of value -0.08 for rounds 1 to 10 and to -0.14 for rounds 11 to 20, a non significant difference at the 5% level. We have not explored the reason why these participants report an extraction level lower than the socially optimal value. The assumption we can make is that it is a way to slow down the increase towards the maximum extraction.

*11. I did not find a discussion of the limitations of the authors approach in the conclusions section. From my previous review: the authors could discuss some obvious limitations of their study and point out directions for future research. As far as I can see, the main limitations of the study are: (1) there is no control condition without information provision, which would correspond to the standard nowadays (most people do not know how much their neighbors extract or what the average in their neighborhood or their village/ city is). (2) Even if free-riders did not disclose their extraction level, given the small groups (n =4) participants could infer the number of free riders from average or even own earnings in a round*

In the revised version we added the limitation about the group size (it was formulated as an interesting point for further investigation). We have now mentioned also the limitation about the reference treatment that could have been a treatment without information at all.

**Reviewer #4**

*The review work is mostly satisfying.*

*However, I felt that limitations of the research is not yet fully explained such as the implementation of the mechanisms author propose in a dynamic environment shall be a very good idea for extension. However, for a first case study author have taken the static framework to make it easier and allows a better understanding of the behavioral dimension of the effects induced by these voluntary sharing mechanisms, which is understandable. Therefore, I think, author should still go ahead and report this.*

In the current version of the paper we have mentioned two possible limitations : the benchmark that could have been a treatment without information at all and the group small group size that can help players to infer individual extractions even if not disclosed.

We have also written a sentence about the possible extension of this research question in a dynamic framework.
