# Supplementary material for: Contrasting effects of information sharing on common-pool resource extraction behavior: Experimental findings
Source: PLoS One. 2020 Oct 7;15(10):e0240212. doi: 10.1371/journal.pone.0240212 (PMC7540899; doi:10.1371/journal.pone.0240212)
Supplement: Attachment — Submitted filename: Reviewer 3.docx [file pone.0240212.s006.docx]

***# Reviewer 3***

We would like to sincerely thank you for your careful reading and the suggestions you made to improve our article.

*For better readability of the results section, please consider adding references to the estimates and the corresponding statistics in the regression tables that you mention in the text in the text as well. Otherwise, the reader will not be able to follow how you used the regression models to test for significant differences etc.*

We have made the changes you suggested and we agree that results are more easily read this way.

*Although I think the text is pretty good in terms of language, it could benefit from having it read once more by an editing service or native speaker.*

We paid a native English speaker to read and improve the text of the article, as we had committed to do. We have to admit that now the paper is more fluid and enjoyable to read.
